# Digital technologies in dairy cattle breeding to improve the reproductive function of cows and heifers: A case study in Northern Kazakhstan

**DOI:** 10.14202/vetworld.2024.2385-2397

**Published:** 2024-10-31

**Authors:** Rashit Uskenov, Saltanat Issabekova, Aizada Mukhanbetkaliyeva, Orken Akibekov, Fariza Zhagipar

**Affiliations:** 1 Department of Technology of Production and Processing of Animal Products, Faculty of Veterinary and Animal Husbandry Technology, Saken Seifullin Kazakh Agrotechnical Research University, Astana, Kazakhstan; 2 Department of Veterinary Medicine, Faculty of Veterinary and Animal Husbandry Technology, Saken Seifullin Kazakh Agrotechnical Research University, Astana, Kazakhstan; 3 Department of Microbiology and Biotechnology, Faculty of Veterinary and Animal Husbandry Technology, Saken Seifullin Kazakh Agrotechnical Research University, Astana, Kazakhstan; 4 Joint Kazakh-Chinese Laboratory for Biological Safety, Faculty of Veterinary and Animal Husbandry Technology, Saken Seifullin Kazakh Agrotechnical Research University, Astana, Kazakhstan

**Keywords:** bolus, cows, digital technology, reproductive function, smaXtec

## Abstract

**Background and Aim:**

In some countries, the application of digital technologies in dairy cattle breeding is still under development. This study aimed to investigate the use of digital technologies in dairy cattle breeding to improve the reproductive function of cows and heifers in three northern regions of Kazakhstan.

**Materials and Methods:**

This study explores the application of Austrian smaXtec bolus sensors, which enable the daily monitoring of the reproductive functions of cows and heifers in livestock. To control indicators of reproductive function in Simmental and Holstein-Friesian cattle breeds, a series of experiments were conducted before and after the introduction of boluses in the rumen.

**Results:**

It was established that the application of smaXtec boluses increases milk yield in 305 days, the percentage of conception in the first insemination and in cows with up to three inseminations, the duration of dry secretion, and the percentage of calve output per 100 heads. Moreover, the use of smaXtec boluses reduced the insemination index, duration of the calving-to-conception interval (open days), reproductive rate, and percentage of abortions and culls due to gynecological problems.

**Conclusion:**

The use of smaXtec boluses allows farmers and veterinarians to determine indicators, such as the period of sexual heat in livestock and diseases, in a timely manner and to increase the efficiency of feeding and controlling drinking cycles. Moreover, the application of smaXtec boluses minimizes labor costs associated with collecting data on indicators of reproductive function in cows and heifers and increases accuracy.

## Introduction

Cow milk production and quality are the most important elements of successful dairy farming. A decrease in milk production and quality reduces farm profits and indirectly affects the reproductive quality and energy balance of the cow. Globally, annual milk production by dairy cows has been increasing almost linearly for many decades [1]. Continuous selection toward higher milk yields, along with improvements in management, housing, feeding, and veterinary care, has led to high-yielding dairy cows that produce more than 35,000 kg of milk per year [2]. Digitalization is widely used to modernize milk production and quality, which implies the application of digital, information, and communication technologies as a new level of development for dairy cattle breeding [3]. The use of technology to monitor behavioral, physiological, or production markers for individual animal diseases, estrus, and comfort is becoming increasingly popular [4]. Technology can monitor indicators such as ovulation time, chewing time, walking activity levels, temperature, and milk yield [5].

Currently, highly mechanized technological processes of housing, feeding, milking, and breeding of dairy cattle are successfully used in the USA, France, the Netherlands, Germany, and other countries. This has significantly increased the milk productivity of cows, leading to improved farm economic efficiency [6]. Innovative technologies have replaced many labor-intensive processes, such as milking cows (40% of total labor costs), feed distribution (30%), and manure removal (15%) [7]. Recently, an algorithm was created to remove drinking points from the reticular temperature and link the reticulorubic fermentation temperature to the vaginal temperature using the reticulorubic temperature (bolus sensor). This algorithm quantifies the relationship between vaginal and reticular temperatures and enables reliable continuous online estimation of cow body temperature using a rumen bolus. Boluses can measure both temperature and pH. Wireless boluses can transmit data every 10 min and can be stored in the cloud or on a personal computer [8]. The bolus developed by smaXtec (SmaXtec, Graz, Austria) is a simple tool that allows dairy farmers to easily monitor cow health, fertility, and lameness. Boluses are administered orally, like magnets, and then deposited in a grid where they will remain safe throughout the cow’s life. Measuring cows’ temperature and activity levels can detect numerous problems that might otherwise go undetected by visual observation [9]. Woodward *et al*. [10] studied the effects of ambient temperature and heat shock in 443 cows on three farms in New Zealand using smaXtec boluses. Their results could predict a high proportion of heat stress cases (sensitivity 34%–68%) but were not sufficiently discriminant, also predicting a high number of false positives (accuracy only 9%–27%). According to Woodward *et al*. [10], bolus data require further investigation with the inclusion of additional variables related to heat shock, such as respiratory rate, age, and body weight. In the dairy cattle industry, the application of sensory technology is a rapidly developing area [11]. A study by Khanal *et al*. [12] published in 2010 on trends in dairy cattle technology did not mention methods of monitoring cattle using internet technology. Similar studies by Iwasaki *et al*. [13] and El Bilali *et al*. [14] favor continuous monitoring of cows as one of the most important technologies for livestock production. A review of the literature using automated methods to track cows for mastitis identified the relationship between rumen temperature, incidental infections, and the occurrence of mastitis. This review showed that sensor data for assessing mastitis status are sufficient to prevent the occurrence of the disease based on rumen temperature readings [15]. Antanaitis *et al*. [16] evaluated the effect of temperature and humidity index on reticulorubic parameters using smaXtec such as temperature, pH, rumination index, and walking activity level of cows showed that the effect of heat shock on reticulorubic parameters increased the risk of acidosis and activity level of cows. Heat shock had a negative effect on reticulorubic pH, temperature, and rumination index. Higher temperatures and rumination index (≥72) increased the risk of rumen acidosis and decreased physical activity in cows. The study by Abdrakhmanov *et al*. [17] shows that regular use of SmaXtec in herd management increased health by 25%, increased growth by 20%, and significantly reduced herd disease.

This study aimed to comprehensively investigate the effects of smaXtec boluses on cow health, disease prevention, and milk yield of cows and heifers of the Simmental and Holstein-Friesian breed to increase productivity, increase the mating period, improve reproductive quality, and temporarily identify deviations in cow health and body condition, which may allow to strengthen the control of dairy production.

## Materials and Methods

### Ethical approval

All animal activities were carried out in compliance with high biosafety and animal welfare standards. All protocols were implemented in accordance with the Ethical Guidelines for the Use of Animals in Research (2019) National Committee for Research Ethics in Science and Technology (NENT) [18].

The Animal Ethics Committee of the Faculty of Veterinary Medicine and Animal Husbandry Technology of the NCJSC “S. Seifullin Kazakh Agrotechnical Research University” (KATRU), Astana, Kazakhstan (protocol No.1), approved the care and use of laboratory animals.

### Study period and location

The study was conducted from February 2018 to December 2020 at the Faculty of Veterinary and Animal Husbandry Technology, S. Seifullin Kazakh Agrotechnical Research University and at the five farming facilities in Northern Kazakhstan.

### Selection of animals and installation of the SmaXtec system

The base farms selected were five dairy and dairy-meat cattle breeding enterprises: “Family farm” LLP (Akmola region, Kazakhstan) and “Olja-Sadchikovskoye” LLP (Holstein breed) (Kostanai region, Kazakhstan), “Mambetov and Company” LP (Simmental breed) (North Kazakstan region, Kazakhstan), “Sartai-agro” LLP (Simmental breed) (Akmola region, Kazakhstan), and “Farmer” farm (Simmental breed) (Akmola region, Kazakhstan).

In all base farms, economic activity was analyzed and genealogical analysis of herds for entry into the SmaXtec bolus system was carried out, and zootechnical analysis of fodder and milk productivity of dairy cows was carried out. Table-1 shows the herd structures in the base farms.

**Table-1:** Herd structure in base farms.

Gender and age groups	“Olja-Sadchikovskoye” LLP	“Family farm” LLP	“Mambetov and Company” LP	“Farmer” farm
Cows	730	32	260	-
Heifers	157	13	330	-
Heifers over 1 year old	246	-	105	96
Heifers under 1 year old	190	5	-	-
Bulls < 1 year old	-	1	-	-
Total	1323	51	695	96

The largest herd was concentrated in “Olzha-Sadchikovskoye” LLP. There was a slight growth dynamic of the milking herd, but in general, the herd remained at the level of 2018. In 2019, “Mambetov and Company” LP purchased 100 more heifers for herd repair, and calving started in August and in October 2019, control milking of 260 cows was carried out. “Family farm” LLP decreased the number of livestock, as this year’s litter of 18 calves, five heifers, and the remaining were bulls sold at the end of the milking period.

Artificial insemination was used in all enterprises. Insemination of heifers implanted with boluses was performed based on reports received from the system (experimental group). The remaining animals without boluses (control group) were inseminated through double observation and sexual heat determination by specialists in the field of animal insemination.

Since 2018, in “Olzha-Sadchikovskoye” LLP and “Mambetov and Company” LP, 200 heads each were selected, and boluses SmaXtec Basic 190 heads and 10 heads were installed with a determination of the pH level in the rumen. In “Family farm,” LLP boluses were in all 50 heads of heifers, and in “Farmer” farm, 43 heads of heifers were installed with boluses that were suitable by weight and age.

All animals were subjected to clinical examination before bolus administration.

### Feed and animal nutrition

Cows on all farms were fed twice daily: in the morning and evening, and concentrated fodder was also fed at lunchtime. Feed distribution was performed on the feed table.

Samples of forages were taken from the base farms to determine their chemical composition and nutritive value. During the organoleptic evaluation of fodder, we found all fodder to be of good quality, and the chemical composition of the fodder is presented in Table-2.

**Table-2:** Chemical composition of fodder according to natural moisture content.

Feed type	Humidity	Dry matter	Crude protein	Crude fat	Crude fiber	Sugar	Carotene (mg)	Calcium (g)	Phosphorus (g)
Wheat hay	16.3	83.7	9.4	2.3	30.9	0.9	20.0	7.8	2.0
Wheat straw	17.8	82.2	5.9	2.2	35.2	-	1.7	2.6	1.2
Haylage	51.2	48.8	6.1	1.2	1.3	0.2	13.1	4.3	1.7
Corn silage	71.8	28.2	5.9	0.8	0.9	0.7	6.9	2.4	0.6
Mixed fodder	12.0	88.0	22.4	3.4	7.6	1.3	0.0	2.2	3.6
Barley grain	11.6	88.4	11.5	2.4	3.5	0.9	-	2.0	4.0
Pea grain	10.2	89.8	19.3	3.9	2.9	3.7	-	1.9	3.79
Oat grain	10.5	89.5	10.3	5.0	3.1	1.4	-	1.5	3.3

Table-2 shows that all forage prepared on the farm was close to the standards of nutrients. In feeding, cows were offereed factory premixes with known composition and nutritional value. The root and tuber crops in the diet are presented as fodder carrots and potatoes. Feeding rations for dairy cows are presented in Table-3.

**Table-3:** Ration of dairy cows of “Olzha-Sadchikovskoye” LLP.

Feed type	Quantity (kg)	Dry matter (kg)	Metabolizable energy	Digested protein (g)
Wheat hay	2.5	2.05	15.25	100
Crushed barley	2.5	2.3	25.75	200
Crushed peas	1	0.90	10.8	192
Corn haylage + silage	30	6.83	66.6	780
Feed carrots	3	0.36	6.6	24
Fodder potatoes	3	0.66	8.48	30
Salt	0.03	-	-	-
Premix	0.18	-	-	-
Total	42.2	13.1	133.48	1326

As can be seen from Table-3, the type of feeding of dairy cows adopted by the enterprise is voluminous (in terms of dry matter 75.6% of nutrients due to voluminous fodder and 24.4% due to concentrated fodder). The metabolizable energy of the ration in “Olzha-Sadchikovskoye” LLP was 133.48 MJ, with a digestible protein of 1326 g. When calculating the need for the level of productivity that cows have, a small need for dry matter (approximately 2.5 kg) is noticed; by adjusting the ration, this deficiency will increase other nutrients contained in the ration.

### Statistical analysis

The mean value and statistical error were calculated using Microsoft Excel 2010 (Microsoft Office, Washington, USA). Repeated measurement analysis of variance was used to determine differences between the numbers of larvae at different stages of invasion and between antibody levels. p < 0.05 was considered statistically significant.

## Results

### pH and milk productivity of the cows

**Figure-1:**
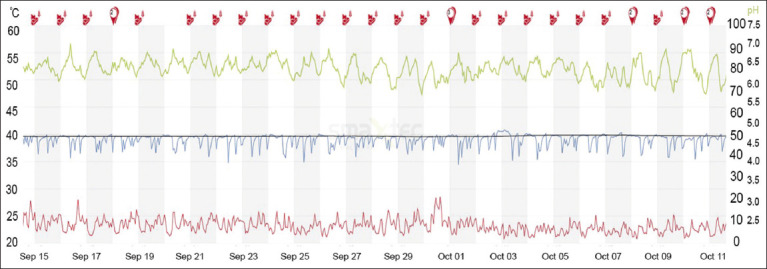
Rumen acidity level in cow No. USA72099496 from “Farmer” farm.

The influence of acidity on cows’ milk productivity was measured using boluses with pH detection. Considering a cow as an example, it is possible to consider the dynamics of changes in indicators measured by the bolus. In cow No. USA72099496 from “Farmer” farm, data were collected on 71 days of lactation. During the month, there were jumps in rumen acidity on average by 0.70 (Figure-1).

As mentioned in Figure-1, for 1 month, the system was alerted daily to reduced feeding efficiency. According to Gasteiner *et al*. [19], acidity increase or decrease during the day was not very severe. It is more important not to allow sharp jumps in acidity over several days at the same time; the acceptable norm of pH change is 0.40–0.60. When analyzing milk productivity in the same cow, we observed the following dynamics: on the 71^st^ day of lactation, a sufficient level of average daily productivity for this breed (25.7 kg. and already on the 101^st^ day, that is, the 3^rd^ month of lactation, there was a sharp decrease of more than 10 kg of milk, which amounted to 15.4 kg. The quantitative milk composition in the 2^nd^ month of lactation was within the norm for the breed: fat, 3.7%, and protein, 3.5%. By the 101^st^ day of lactation, the fat amount was lower than protein: fat, 3.38% and protein, 4.73%. When determining the number of somatic cells on the 2^nd^ month of lactation, this indicator was within the physiological norm – 268,000/mL, at the next control milking, the number of somatic cells was very high – 970,000/mL, which indicates inflammatory processes in the udder during this period, which is most likely associated with a general decline in immunity against the background of incorrect feeding. According to Bach *et al*. [20], pH level and feeding regimen are directly related. DelCurto-Wyffels *et al*. [21] used mono-feeds with pH regulators found that a lower pH reduced dry matter intake compared to a higher pH. At rumen pH values below 6.2, it suppresses the rate of digestion, favors the growth of lactic acid-producing bacteria, and suppresses the growth of cellulolytic, hemicellulolytic, and pectolytic bacteria.

**Figure-2:**
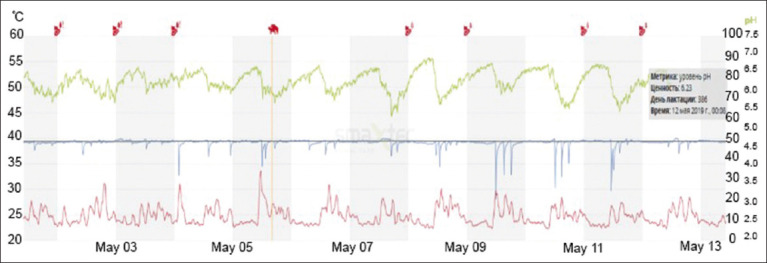
Rumen acidity level in a cow in “Family farm” LLP.

The same results were obtained when establishing the relationship between rumen pH and milk productivity in “Family Farm” LLP; only the change in acidity and somatic cell count varied during the day. In “Family Farm” LLP, monthly control milkings were conducted, which showed that milk productivity was at the level of 18.3 ± 2.51 kg, percentage of fat 3.72% ± 0.05% and protein 3.22% ± 0.03%, and the number of somatic cells 213.7 ± 7.32 thousand/mL. However, even on this farm, there were alerts about the incorrect feeding of cows (Figure-2).

Daily changes in rumen acidity lead to various diseases, from ketosis to laminitis. Thus, during the control milking in September, the cow’s milk in the morning milking showed a somatic cell count within 136,000/mL, which is an indicator of a healthy udder, whereas in the evening, this indicator was equal to 566,000/mL, which indicates the beginning of mastitis.

The biometric processing of milk productivity and acidity in Olja-Sadchikovskoe LLP showed that the average daily milk yield of cows in October was 16.9 ± 2.41 kg, while for cows with reduced acidity, it was 13.4 ± 1.62 kg. When calculating the correlation coefficient between pH level and average daily milk yield for the cows, an average positive relationship of 0.33 was determined, indicating that milk productivity decreases with decreasing acidity in the rumen. It can be concluded that high rumen acidity negatively affects milk productivity.

**Figure-3:**
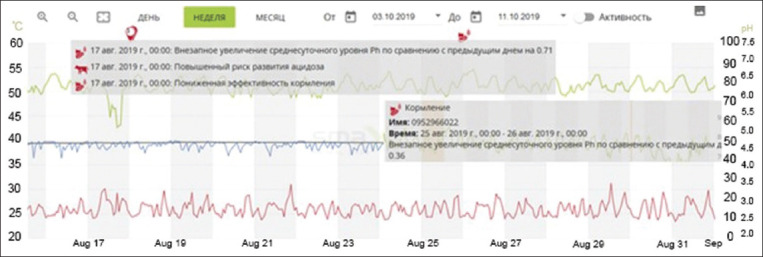
Changes in acidity of cow No 095296622 “Mambetov and Company” LP.

The following research results were obtained from “Mambetov and Company” LP. During control milking in August, animals with increased fat and somatic cell contents in their milk were detected, indicating a violation of digestive processes and the presence of inflammatory processes in the udder. The fat content in these animals was 6.24% and the number of somatic cells was 1,376,000/mL. These changes in milk also converged with the data obtained from boluses (Figure-3).

As shown in Figure-3, fluctuations in rumen acidity were observed in the days before and after the control milking, which ultimately affected milk composition as well as milk yield. A few days before the control milking, there was a report of decreased feed efficiency in this cow. Since the bolus with pH measurement is put only on 10% of cows, other cows of the milked herd experienced the same problems, as evidenced by the average amount of fat in the herd 5.09%, and the average daily milk yield was 12.3 kg. According to the obtained indicators, work was performed to adjust feeding and body condition score (BCS) fatness, and the animals were overfed. It was necessary to reduce the average feed intake and fatness of milked cows to an index of 3.5–3.75 points.

After feeding adjustments, control milking was carried out in September. According to the results, no cows with a violation of the quantitative and qualitative composition of milk were detected. Thus, according to the results of control milking, the average daily milk yield of cows amounted to 19.91 kg, which is 7 kg more than the previous month. The fat index was 4.00%, protein was 3.22%, and somatic cell count was 159,000/mL. These indicators are within the norm for the Simmental breed.

Milk productivity and quantitative and qualitative indicators were analyzed in Holstein–Friesian cows. The milk yield was 16.9 ± 2.41 kg, fat percentage 3.9 ± 0.02%, and protein 3.4 ± 0.05%. In general, we can say that all other indicators were within the norm for the Holstein-Friesian breed. In addition, the udder health index (somatic cells) was within the normal range of 187.3 ± 5.95 thousand/mL.

According to the rumen pH bolus data, the pH level depends on the feeding mode and diet. The relationship between pH and milk productivity was positively correlated at 0.33. Boluses were allowed to adjust the mode and time of cow feeding and to improve milk production indicators, while the number of somatic cells helped predict the onset of mastitis.

### Bolus use and reproductive control

Animals with Smaxtec boluses were monitored to determine the stage of arousal based on movement activity and calving time (Table-4).

**Table-4:** Results of the cow excitement stage in the model farms (%).

Farm	n	Months				

		January	February	March	April	May
“Family farm” LLP	30	10	6.6	16.6	23.3	25.3
“Olja-Sadchikovskoe” LLP	200	15	10	25	30	30
“Mambetov and Company” LP	164	8.5	7.3	15.2	18.2	21.2
“Farmer”	96	11	5.8	16.3	15	17

The data in Table-4 show that the manifestation of sexual hunting in cows in January and February ranged from 6.6% to 15%. Since March, movement activity in animals has increased from 15.2% to 30%. Of the animals that showed movement activity (n = 103), 57 cows (55.3%) were inseminated. Thus, 23 cows with active movement were registered in the period of 1 week, among which 11 (47.8%) showed signs of heat and sexual desire and were inseminated. In the other animals, heat, genital hyperemia, and sexual hunting were not determined. The activity of animals is attributed to the ferrying of animals during mass treatments, feeding, and genital diseases, which prevent fruitful insemination.

Table-5 presents the results of determining the immobility reflex by 2-time observation of the manifestation of the stage of sexual arousal in animals in comparison with reports from the SmaXtec system on the manifestation of puberty in cows.

**Table-5:** Results of determining puberty in cows using the observation method and SmaXtec system.

Method of determination	n	Characteristics	Definition of sexual hunting
Two-times- observation method	30	Arousal, heat, and sexual desire	56.6
SmaXtec system	30	Mobility and sexual desire	83.3

The results show that the SmaXtec system with established herd management improved by 26.7% the detection of cows with signs of sexual desire, thus increasing the fertilization rate and the percentage of pregnant cows. According to the study of Todorović *et al*. [22], the value of conception in cows from the experimental group with smaXtec bolus was 61.54%, whereas in cows of the control group, the fertilization rate was 48.00%. The results of the study indicate that the investigated digital technology can significantly increase the fertility of inseminated cows and thus contribute to the reproductive efficiency of the herd.

The change in temperature was used as a parameter to determine the onset of labor in the cow. To obtain more accurate data on onset of labor, the data of inseminated cows tested for pregnancy were collected. The SmaXtec system indicates a tentative calving date after the diagnosis of pregnancy. The SmaXtec system detects the calving time when the body temperature drops 1 day before parturition. The results are presented in Table-6.

**Table-6:** Calving-time determination by the SmaXtec system.

Day before calving	n	Gestation period (days)	Temperature	SmaXtec	

				n	%
1–2	16	281 ± 4.3	38.1 ± 0.4	13	81.3
3–4	15	279 ± 5.1	39.2 ± 0.3	5	33.3

As shown in Table-6, if the inseminated cows tested for pregnancy were correctly recorded, the SmaXtec system confirmed the reliability of information on the onset of labor in 81.3% of cases. This system increased the number of detected animals with puberty by 26.7% and determined the calving time in 81.3% of cows.

Because all cattle gave litter, a comparative analysis was carried out to study the reproductive function of the breeding stock on four experimental farms (Table-7).

**Table-7:** Comparative analysis of the reproductive function of breeding stock on four experimental farms.

Indicator	Control group	Experimental group	Standard value
Total number of heads	307	240	-
Average age at first insemination of heifers (months)	19.1 ± 3.9	18.5 ± 3.4	18–19
Fertilization rate at the first insemination of heifers (%)	62.7	73.2*	>60%
Insemination index	1.70 ± 0.8	1.3 ± 0.4	1.2
Calf yield per 100 calf (%)	94.9	95.2	More than 95%
Average age at first calving (months)	28.1 ± 5.2	27.5 ± 4.3	26–28

* P < 0.05

Table-7 shows that indicators of the reproductive function of heifers with boluses on farms correspond to normative values. The implantation of boluses reduced the average age of the first insemination of heifers by 4.8%, the insemination index by 23.5%, and the average age of the first calving from 2.1%. In addition, bolus application increased the fertilization rate at the first insemination of heifers by 10.5% and calf yield by 0.3%. The difference in calf yield between the control and experimental groups was insignificant because it depended on many factors, especially feeding.

Thus, although indicators of reproductive function of the breeding stock in both groups corresponded to normative values, the experimental group was more efficient because of the use of boluses in the stock. The control of monthly calf litter on dairy farms is aimed at obtaining the same number of calves per month [23]. In addition to controlling the monthly calf litter, farmers must balance the amount of milk produced, which should be stable at a certain level during the calendar year [24]. Part of the offspring of the remaining heifers completed the first third of lactation. Then, the heifers were inseminated. The first 50 animals that completed the first 100 days of current lactation (25 head with and without boluses) were selected for the experiments. Supplementary T1 presents the reproductive performance of heifers with “Mambetov and Company” LP before and after bolus implantation. Implantation of boluses in “Mambetov and Company” LP increased milk yield per 100 days by 2.1%, fertilization rate at the first insemination of first heifers by more than 23.5%, and insemination rate of first heifers at the number of inseminations up to three increased by more than 7%. In addition, bolus implantation decreased insemination index values by 17.3%, the number of days of fertilization by 24 days, the interbreeding interval by 23.9 days, and the reproductive rate by 11.8%.

**supplementary Table-1:** Indicators of reproductive function of heifers of “Mambetov and Company” LP before and after implantation.

Indicator	Control group	Experimental group
Milk yield per 100 days (kg)	2080 ± 52.3	2123 ± 37.8
Insemination index	1.62 ± 0.8	1.34 ± 0.2
Passing days	102 ± 17.2	78 ± 8.4
Fertility at first insemination of heifers (%)	43.2	66.7
Inseminated heifers with up to three inseminations (%)	90.2	97.1
Calving interval, days (estimated)	372.4 ± 21.4	348.5 ± 19.4
Reproduction rate (estimated)	1.02	0.9

According to Armengol *et al*. [25], farmers and scientists need to consider a more detailed list of indicators of reproductive function to ensure correct control over the fertility status of heifers and cows on farms. This will undoubtedly enable them to control animal reproduction more effectively.

Supplementary T2 presents the reproductive function indicators of cows at “Olzha-Sadchikovskoye” LLP before and after bolus implantation. The results of bolus implantation in “Olzha-Sadchikovskoye” LLP increased milk yield for 305 days by 8.8%, fertilization index at the first insemination by more than 20%, percentage of inseminated cows at three inseminations by 6.6%, duration of dry period by 4.5 days, and calf yield per 100 head by 0.5%. Moreover, implantation of boluses reduced values of indicators such as insemination index by 0.43, inter-insemination interval by 26.5 days, opening days by 9.1 days, reproductive capacity coefficient by 0.3, abortion rate by 0.6%, and culling rate for gynecological problems by 4.3%. According to the results, it was established that “Olja-Sadchikovskoye” LLP is one of the most highly productive farms in the Republic of Kazakhstan, as it produces 6000–7000 kg of milk. The insemination index of cows in the control group exceeded the upper limit, indicating problems with the reproductive function of cows on the farm. In addition, bolus implantation reduced the percentage of culling due to gynecological problems by half. Thus, bolus implantation increases the accuracy of cow fertility control on farms. At the “Family farm” LLP, boluses were implanted in all the cows due to the small number of cows (30 dairy cows). The reproductive function indicators before and after bolus implantation are presented in Table-8.

**supplementary Table-2:** Indicators of reproductive function of cows on the farm “Olzha-Sadchikovskoe” LLP before and after the introduction of boluses.

Indicator	Control group	Experimental group
Milk yield in 305 days (kg)	5836.8 ± 125.25	6355.6 ± 125.6
Insemination index	1.95 ± 0.2	1.52 ± 0.1
Fertility at first insemination (%)	23.7	43.4
Inseminated cows with up to 3 inseminations (%)	88.1	94.7
Calving interval (days)	401.6 ± 2.68	375.1 ± 3.45
Interlactation period (days)	56.5 ± 1.20	61.0 ± 1.22
Open calf day (days)	101.0 ± 3.07	91.9 ± 3.12
Calves per 100 head (%)	93.8	94.3
Reproduction rate	1.05 ± 0.22	1.02 ± 0.27
Abortion rate (%)	4.3	3.7
Culling due to gynecological problems (%)	8.4	4.1

**Table-8:** Indicators of reproductive function of cows of “Family farm” LLP before and after the introduction of boluses.

Indicator	Control group	Experimental group
Milk yield in 305 days (kg)	3823 ± 274	4057 ± 274
Insemination index	1.81 ± 0.09	1.33 ± 0.05
Fertility at first insemination (%)	37.3	52.6
Inseminated cows with up to three insemination (%)	84.3	96.3
Calving interval (days)	400.4 ± 25.68	379.0 ± 23.18
Interlactation period (days)	57.5 ± 1.21	63.0 ± 1.23
Calving ease (days)	114.3 ± 7.1	99.7 ± 4.3
Calves per 100 heads (%)	94.3	95.8
Reproduction rate (%)	1.08 ± 0.23	1.04 ± 0.11
Abortion rate (%)	3.7	3.3
Culling due to gynecological problems (%)	5.8	2.7

According to the results, bolus implantation increased the values of some indicators, such as milk yield at 305 days by 6.1%, fertilization rate at first insemination by 15.3%, percentage of inseminated cows at up to three inseminations by 12%, interaction period by 5.5 days, and calf yield per 100 heads by 1.5%. Moreover, the implantation of boluses reduced the values of some indicators, such as the insemination index by 0.48, intercalf interval by 21 days, duration of open calf period by 14.6 days, reproductive capacity factor by 0.04, abortion rate by 0.4%, and culling rate for gynecological problems by 3.1%.

Based on the results obtained, boluses increase milk yield. This means the infrastructure and feed base in “Family farm “ LLPs have been improved. According to veterinarian recommendations, implanting boluses, which reduces the insemination index, allows physicians to more accurately determine the time of estrus. Thus, veterinarians can ensure timely insemination of cows [26].

The reproductive indices of Simmental heifers were analyzed before bolus implantation (Table-9).

**Table-9:** Indicators of reproductive function of Simmental heifers before the introduction of boluses.

Indicator	Obtained value (n = 65)	Optimal value for the heifers	Normal range
Insemination index	1.5 ± 0.3	1.2	2 and more
Fertilization during the first insemination of heifers (%)	47.1	65–70	<60
Inseminated heifers with up to three insemination (%)	88.6	More than 90	<90
Average age of heifers at first insemination (months)	18.3 ± 3.7	16–19	More than 20
Average age at first calving (estimated) (months)	27.4 ± 4.1	24–28	More than 28
Abortions (%)	1.53	<5	More than 5

Table-9 shows that most indicators are within the normal range. However, the values of some indicators, such as the insemination index, fertilization percentage at the first insemination, and insemination of heifers for up to three inseminations, were outside the norm. This means that the farm lacks an organized process for detecting the hunting period in animals and artificial insemination technology.

Based on an analysis of the production technology, it was found that the heifers were overfed. This result was confirmed by the fatness score (BCS), which was >3.75 [27]. As a rule, cows’ reproductive performance is directly related to their feeding. Therefore, overfeeding the productive type of the Simmental breed represents a risk of a faster transition to beef cattle than in other breeds [28, 29].

In our study, feeding on all farms was optimized using smaXtec boluses. Based on the messages received from the boluses, farmers adopted changes in feeding technology and nutrient balance in the ration. As a result, the heifers with the service age achieved a BCS value of 3.5.

### Microclimate on the farm and its impact on cow productivity

The formation of microclimate in animal rooms depends on several conditions: local climate, thermal and humidity state of building envelopes, air exchange or ventilation, heating, sewerage, and lighting, as well as the degree of heat production by animals, density of their accommodation, housing technology, and daily routine [30].

This study conducted control measurements of the microclimate parameters to determine the full reliability of the SmaXtec Climate Sensor data. For this purpose, control measurements of microclimate parameters were conducted at the farms of “Olzha-Sadchikovskoye” LLP and “Mambetov and Company” LP. These control data fully corresponded to the transmitted data of the SmaXtec climate sensor. According to the results of our study, the animals well tolerated low air temperatures and accordingly changed indicators of air humidity; here, it is necessary to note a very important component of the microclimate of the speed of air movement, which corresponded to the norm and did not exceed 0.2 m/s.

**Figure-4:**
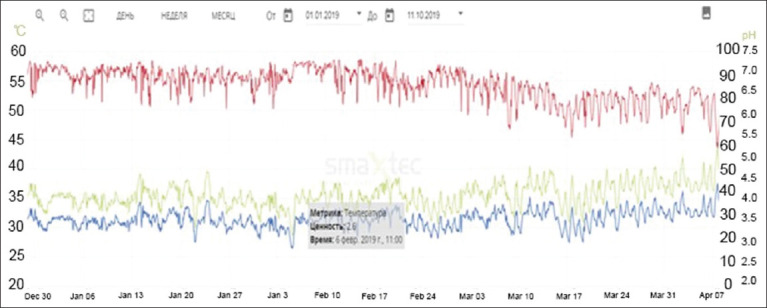
Graphical data of temperature and humidity level fluctuations obtained from SmaXtec Climate Sensor (“Olzha-Sadchikovskoye” LLP).

As shown in Figure-4, the data are transferred in the form of a graph, with three curves indicated by blue, red, and yellow colors. Blue indicates the air temperature, red indicates the humidity in the room, and yellow is the index of the temperature-to-humidity ratio, the so-called THI index.

Temperature indicators were reduced to 8°C on February 06, 2019. Accordingly, there was a decrease in milk productivity (average daily milk yield per 1 cow – 12.3 kg, fat – 5.09%, protein – 3.00%, and number of somatic cells – 231 thousand/mL) cold stress, but on the following days with almost the same indicators of milk productivity indicators came to normal (adaptation).

Later in the summer, we conducted a study to clarify the influence of high temperature and air humidity on milk productivity. For this purpose, we monitored data obtained from the SmaXtec Climate Sensor as usual.

**Figure-5:**
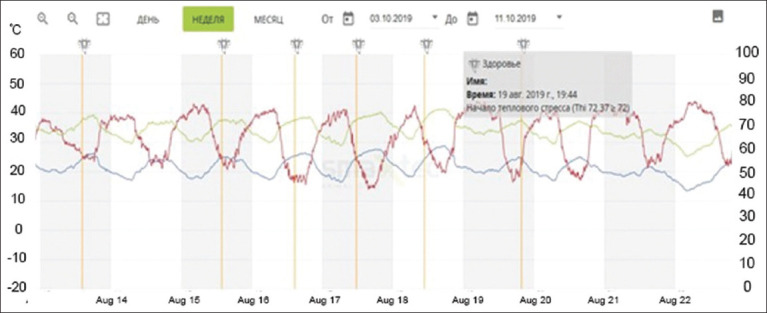
Climate sensor indicators in “Mambetov and Company” LP.

Thus, in August 2019, in “Mambetov and Company” LP, a high air temperature of 27°C, exceeding the recommended norm (23.5°C), was recorded, which contributed to heat stress. According to the observations, temperature and humidity fluctuations affected the indicators of milk productivity, with a decrease in air temperature and changes in humidity in the air environment (Figure-5).

The data obtained from the climate sensors were continuously analyzed and compared with productivity indicators. From the temperature curve, we can see that it increased above the norm in the last few days of May and fell at high ambient temperatures (norm 4–23°C). During the same period, indoor humidity also tended to increase. However, THI was within the normal range, heat stress occurs at THI value ≥72, if THI ≥78, it is medium, and if THI ≥82, it is severe.

In this farm, the average daily milk yield per cow in August was 12.3 kg fat, 5.09% protein, 3.0% somatic cell count, and 231,000/mL (data of control milking August 20, 2019). In September, there was a repeated control milking where the data were normal: average daily milk yield per cow: 19.91 kg, fat: 4.0%, protein: 3.22, and number of somatic cells 159,000/mL. These data show that animals tolerate low temperatures better than high temperatures. Based on the analysis of sensor readings, we found that the effect of heat stress on cows’ organisms begins to appear at indoor air temperatures above 25°C. The data are confirmed by Jo *et al*. [31], who reported that summer weather in Korea had a negative effect on milk yield, milk fat, milk protein, somatic cells, rumen activity, and rumen temperature of Holstein cows. In addition, a correlation was found between the degree of heat stress experienced by Holstein cows based on parity. Antanaitis *et al*. [32] used the RumiWatch sensor (RWS; ITIN + HOCH GmbH, Fütterungstechnik, Liestal, Switzerland) and showed that the activity of cows increased by 11.75% at THI >78 compared to THI <72. The cows at THI >78 had higher blood urea nitrogen levels than the cows at THI 72–78.

Microclimate parameters showed no deviations from the normal and, accordingly, did not affect animal performance or health (milk productivity and cases of animal diseases).

## Discussion

The bolus implantation has been successfully implemented in many countries, such as USA, New Zealand, Australia, Central Europe and Ireland compared with Commonwealth of Independent States countries, digital systems in the field of livestock breeding quickly paid off in other countries. These digital systems allow farmers to monitor the health of cows and heifers in detail using ear chips, collars, and bracelets. Caja *et al*. [33] wrote in their review article that the use of smart systems to track cow health and fertility status is gaining momentum in today’s dairy business.

Our study suggests that such systems are efficient on farms in Kazakhstan, especially smaXtec boluses, which improve the reproductive function of cows. Undoubtedly, this can increase Kazakh farmers’ demand for boluses.

Kirsanov *et al*. [34] investigated several ways to monitor herd health under current conditions. Their work examines the existing health monitoring systems used on dairy farms. Based on this objective, they performed a comparative analysis of health monitoring systems currently available on the market from manufacturers, such as SCR Heatime HR (Allflex, Netanya, Israel), E093 SmartbowEartag (Smartbow GmbH, Weibern, Austria), and smaXtec boluses. They also studied relevant scientific articles and concluded that smaXtec boluses are most effective for health monitoring due to the use of additional climate sensors and pH meters.

Antanaitis *et al*. [35] used a bolus system to measure body temperature and rumen acidity to predict cow fertility. The results show that cows with impaired metabolism have a low fertility status, and boluses can be a tool for controlling that status.

Data from Antanaitis *et al*. [36, 37], a bolus system, were used to support their hypothesis that continuous condition tracking with boluses is an indicator of cows’ health and reproductive status. In accordance with Liang *et al*. [38], the SmartBolus system (TenXSys Inc., Eagle, Idaho, USA) was used to detect disease, heat stress, and general physiological stress. They found that the data obtained from the boluses can be useful for heat stress management and stress selection in resistant animals.

However, the calculation of the payback period of digital technologies in Kazakhstan cattle breeding has been insufficiently studied. Researchers tested a few approaches to control herd conditions in state-of-the-art facilities [34]. The authors conducted a comparative analysis of animal health monitoring systems on the market, such as “SCR - Heatime HR,” “Smart-bow - Eartag E093,” and “smaXtec – boluses.” Based on the results, the researchers concluded that smaXtec boluses are the most effective for monitoring animal health since they can be applied to additional climate sensors and pH meters. However, the authors noted that all animal health monitoring systems are expensive. Russian farmers have refused to use boluses because of high costs. Bykovskaya and Vlasova [39] calculated the payback period of digital technologies used in dairy farming. The authors revealed that applying smaXtec boluses, with an average herd yield of 6000 kg/head, allowed the farmers to pay off the boluses in 2.5 years. Moreover, they investigated the well-known “Dairy Plan” herd management software and the “Lely Vector” robotic milking and feeding system and noted that the reviewed animal health monitoring systems are unavailable in rural areas. They also attributed this to factors such as the lack of access to high-speed internet in one-third of farms, digital literacy among villagers, and low demand for online services.

This is consistent with a previous study by Artyomova and Shpak [40], who pointed out that one of the reasons for reduced milk production and consumption in Russia is the technological lag in dairy cattle breeding from foreign countries. The proportion of dairy farms that use modern technologies and equipment is 10%–15%. Moreover, the infrastructure of new and reconstructed dairy complexes and farms does not always meet modern technological requirements for keeping and feeding highly productive livestock. They also revealed that developing automated control systems in dairy farming increases the intensity of equipment use and reduces labor and material costs. Thus, it allows farmers to enhance their technological effects, thus providing the most favorable conditions for animals. Such activities can increase milk yield by 25%, livestock reproduction by 20%, and reduce the percentage of sick animals. Due to the operational monitoring of the level of milk production and the timely implementation of veterinary measures, it is possible to increase the value of the period of productive use of cows and improve the indicators of the reproductive function of the herd.

Operational monitoring of milk production levels and timely veterinary measures can increase the period of productive use of cows and improve the reproductive function indicators of the herd.

In our study, the application of boluses allowed us to determine the regularity of the negative impact of high milk synthesis on a cow’s physiological health. A cow’s fertility level rapidly deteriorates when the animal spends all its body’s reserves on milk production. The established negative correlation between the indicators of reproductive function in cows confirmed this regularity. Our study reported positive effects of the bolus system, which were conducted under different climatic conditions than those of Kazakhstan, including forage and technological conditions.

Based on the results obtained in our study, during dairy cattle breeding in Kazakhstan, first heifers do not inseminate for the 1^st^ time. The deterioration of the indicators of reproductive function implies a low level of the feed base. Low feed base levels do not meet the needs of animals due to the lack of qualified farm employees who are needed to control feeding and reproduction technology. Our study suggests that boluses are efficient in dairy farming in Kazakhstan because they improve indicators of the reproductive function of cows and heifers. These digital technologies allow farmers to solve many problems, including those not identified by them in the initial stage of the production process. Moreover, the correct implementation of digital technologies under climatic conditions similar to those of Kazakhstan (e.g., Russia, some parts of Asia, etc.) would allow farmers to identify problems with cows in time, thereby avoiding irreversible harm to the animal.

## Conclusion

The use of the Smaxtec system allows us to effectively monitor the health and productivity of animals in real-time. Considering that studies before and after the implantation of boluses with rumen pH measurements are an auxiliary part of the whole milk production process, an average positive relationship between pH level and average daily milk yield was determined. The use of the Smaxtec system increased the number of animals detected at puberty by 26.7% and determined the calving time in 81.3% of cows. Microclimate parameters did not deviate from the norm and accordingly were used for indicators of productivity and health of animals (milk productivity and cases of animal diseases). It is worth noting that animals better tolerate cold stress than heat stress. This suggests that advanced technologies can help dairy farmers effectively control the reproduction and productivity of cows. Additional research is needed for different climatic conditions, dietary regimes, and compositions, including cows and heifers of different ages, activities, and health statuses.

## Data Availability

All data generated or analyzed during the study are included in this article.

## Authors’ Contributions

RU: Designed and supervised the study. SI and AM: Designed and conducted the study. OA: Designed the study, performed statistical analysis, and drafted the manuscript. FZ: Performed statistical analysis and drafted the manuscript. All authors have read and approved the final manuscript.
